# Genome-wide transcriptome reveals mechanisms underlying *Rlm1-*mediated blackleg resistance on canola

**DOI:** 10.1038/s41598-021-83267-0

**Published:** 2021-02-23

**Authors:** Chun Zhai, Xunjia Liu, Tao Song, Fengqun Yu, Gary Peng

**Affiliations:** 1 Agriculture and Agri-Food Canada (AAFC), Saskatoon Research and Development Centre, Saskatoon, SK Canada; 2 Present Address: Syngenta Biotechnology China Co., Ltd, Beijing, China

**Keywords:** Molecular biology, Plant sciences

## Abstract

Genetic resistance to blackleg (*Leptosphaeria maculans*, *Lm*) of canola (*Brassica napus*, *Bn*) has been extensively studied, but the mechanisms underlying the host–pathogen interaction are still not well understood. Here, a comparative transcriptome analysis was performed on a resistant doubled haploid *Bn* line carrying the resistance gene *Rlm1* following inoculation with a virulent (*avrLm1*) or avirulent (*AvrLm1*) *Lm* isolate on cotyledons. A total of 6999 and 3015 differentially expressed genes (DEGs) were identified, respectively, in inoculated local tissues with compatible (susceptible) and incompatible (resistant) interactions. Functional enrichment analysis found several biological processes, including protein targeting to membrane, ribosome and negative regulation of programmed cell death, were over-represented exclusively among up-regulated DEGs in the resistant reaction, whereas significant enrichment of salicylic acid (SA) and jasmonic acid (JA) pathways observed for down-regulated DEGs occurred only in the susceptible reaction. A heat-map analysis showed that both biosynthesis and signaling of SA and JA were induced more significantly in the resistant reaction, implying that a threshold level of SA and JA signaling is required for the activation of *Rlm1*-mediated resistance. Co-expression network analysis revealed close correlation of a gene module with the resistance, involving DEGs regulating pathogen-associated molecular pattern recognition, JA signaling and transcriptional reprogramming. Substantially fewer DEGs were identified in mock-inoculated (control) cotyledons, relative to those in inoculated local tissues, including those involved in SA pathways potentially contributing to systemic acquired resistance (SAR). Pre-inoculation of cotyledon with either an avirulent or virulent *Lm* isolate, however, failed to induce SAR on remote tissues of same plant despite elevated SA and *PR1* protein. This study provides insights into the molecular mechanism of *Rlm1*-mediated resistance to blackleg.

## Introduction

Canola or oilseed rape (*Brassica napus* L.) is one of the most important sources of vegetable oil and livestock feed (meal) in the world, with an estimated global annual production of 70 million metric tons^1–3^. Blackleg, caused by the hemibiotrophic fungal pathogen *Leptosphaeria maculans* (*Lm*) (Desm.) Ces. & de Not. (anamorph *Phoma lingam* (Tode ex. Fr.) Desm.), is one of the most serious diseases affecting canola/oilseed rape production in many parts of the world except China, with average yield losses around 10–20% each year^2–5^. This disease can be a major economic constraint to canola production in Australia, Europe and North America^2,5^, with estimated annual crop losses at > US$900 million in these regions alone^6^.

The blackleg infection often starts with the invasion of cotyledons and younger leaves via stomata or wounds. The fungus then colonizes intercellular spaces between mesophyll cells, causing visible lesions. Once the fungus enters the petioles, it can rapidly spread to stems^7–10^. *Lm* may also infect older plants through the base of stem or the points of leaf attachment^11^. The invasion of cortical tissue in the stem base and subsequent development of collar necrosis usually result in the formation of blackened canker that girdles the stem and causes plant lodging^7–10,12^. Although an integrated strategy is recommended for blackleg management, genetic resistance is the mainstay^13^.

The resistance to blackleg in *B. napus* is often categorized as either seedling or adult-plant resistance. The latter is also referred to as partial resistance that may not prevent lesion development effectively on leaves but can reduce the severity of stem infection. This type of resistance is mediated generally by multiple minor loci and is race non-specific^14–16^. In contrast, seedling resistance restricts infection on leaves, thus preventing *Lm* from spreading into stems via petioles. This type of resistance is controlled by resistance (*R*) genes that interact with the corresponding avirulence (*Avr*) genes in *Lm* in a race-specific ‘gene-for-gene’ manner^14,17,18^. In blackleg-resistance breeding, race-specific *R* genes have been used commonly likely due to its simple genetic determinism and selection procedures^19,20^. However, specific *R* genes tend to be short-lived due to rapid evolution in the *Lm* population and the emergence of new virulent isolates^21–24^. To date, 18 major *R* genes (*Rlm1-11*, *RlmS*, *LepR1-4*, *BLMR1* and *BLMR2*) have been reported from various *Brassica* species^14,25–31^. Among them, *LepR3* and *Rlm2*, have been cloned and proved to be an allelic pair encoding membrane-bound receptor-like proteins (RLP)^32,33^. However, modes of action on how these *R* genes confer resistance to different *Lm* races remain elusive.

During the co-evolution with pathogens, plants have developed a highly sophisticated immune system to protect themselves against diseases. Pathogen-associated molecular pattern (PAMP)-triggered immunity (PTI), which is initiated upon the perception of PAMP (the conserved microbial structures such as bacterial flagellin and fungal chitin) through surface-localized pattern recognition receptors (PPRs), constitutes the first line of defense^34^. Adapted pathogens, however, are able to overcome PTI via secreting virulence effectors into host cells^35,36^. Some plant species, however, have evolved to produce intracellular immune receptors that can recognize specific effectors either directly or indirectly, activating the second line of defense, i.e. effector-triggered immunity (ETI)^34^. Both PTI and ETI may share similar downstream responses, including hormone changes, transcriptional reprogramming and defense-gene activation, but differ in response intensities^37–39^. Following pathogen perception, the amplification and transmission of pathogen-induced signals rely largely on phytohormones^40^. Selected transcription factors (TFs) and associated co-factors integrated within signaling pathways decode the information leading to diverse transcriptional reprogramming^41^. As a result, active plant defense accompanied by the production and accumulation of defense-related proteins are induced to restrict pathogen development^42^. In general, PTI is relatively weak and regarded as a basal defense response that may not stop the spread of pathogens but rather influences the severity of disease caused by adapted pathogens^43,44^. In contrast, ETI is more accelerated and robust, often resulting in a hypersensitive response (HR) around the infection site^45^. Besides the induced resistance that acts locally against infection, ETI may also trigger subsequent transmission of signals to distal, non-infected parts of plant causing SAR^38,46,47^.

Genome-wide transcriptome analysis is considered a useful step to identify the molecular basis for the host defensive system in response to pathogen infection. With the advantage of its high-throughput and high sensitivity, next-generation RNA sequencing (RNA-seq) has become the most promising technology for genome-wide transcriptional study^48–50^. In the past several years, it has been widely applied to investigate plant pathosystems, and the transcriptome sequences generated have greatly improved our knowledge of defence networks in plants. Large-scale transcriptome analyses on *B. napus*–*Lm* interactions have also been reported. For instance, Becker et al.^51^ observed an accelerated activation of plant transcriptome associated with transcripts coding extracellular receptor and phytohormones signaling molecules involved in the interaction of *LepR1* and *AvrLep1* using RNA-seq. Similarly, based on transcriptome analysis, Zhou et al.^52^ delineated a hierarchical gene expression network orchestrated by two allelic *R* genes *LepR3* and *Rlm2*, while Becker et al.^53^ reported that calcium and JA play a central role in *Rlm2-*mediated resistance signaling. A better understanding of regulatory mechanisms underlying respective blackleg *R* genes may facilitate the pyramiding of multiple genes with different modes of action to improve resistance durability. In this study, we analyzed global gene expression in the *B. napus* DH line DH24288, a doubled haploid (DH) line developed from a single spore of *B. napus* cultivar ‘Qunita’ carrying *Rlm1*^54^ in compatible (susceptible) and incompatible (resistant) interactions against *Lm* based on RNA-seq. Additionally, potential SAR induced by *Lm* was also investigated.

## Methods

### Plant material, fungal isolates and growth conditions

The *Bn* DH line DH24288 carrying the blackleg *R* genes *Rlm1* and *Rlm3*^54^ was used as the host plant throughout the study. This DH line was developed from the *Bn* cultivar ‘Quinta’ at AAFC Saskatoon Research and Development Centre, and kindly provided by Dr. G Séguin-Swartz.

Seeds were sown to Sunshine #3 potting mix (SunGro Horticulture, Vancouver, CA) to which 12.5 g l^−1^ Osmocote Plus 16-9-12 (N-P-K; Scotts Miracle-Gro Canada, Mississauga, ON) had been added. Seedlings were grown in 72-well flats in a growth chamber with a photoperiod of 16-h light (approximately 280–575 μmol m^−2^ s^−1^) and 8-h darkness with a day/night temperature at 20/18 °C. The *Lm* isolates 12CC329 (harbouring *avrLm1*) and SC006 (harbouring *AvrLm1*), which are virulent and avirulent, respectively, to DH24288, were cultured on V8-juice agar in Petri dishes with a 12-h photoperiod at 22 °C for about 10 days to produce pycnidiospores as the inoculum.

### Cotyledon inoculation and sample preparation

The collection and preparation of inoculum, as well as inoculation of canola seedlings, followed the protocols described previously^29^ with minor modifications. Briefly, pycnidiospores were harvested from the single-spore cultures of the *Lm* isolates by washing the plates with sterile distilled water, and the spore concentration was adjusted to 2 × 10^7^ spores per ml using a haemocytometer. At about 7 days after seeding, both lobes of a cotyledon were punctured with a sterile needle and inoculated with 10 μl of the inoculum or distilled water (mock inoculation), while the other cotyledon was left un-inoculated. Inoculated plants were maintained in the growth chamber described above.

Seven days post inoculation (dpi), samples were collected from *Lm*- and mock-inoculated cotyledons, as well as from the non-inoculated cotyledons, by punching out discs (0.6 cm in diameter) around the centre of inoculation or cotyledon lobe (non-inoculated). At 7 dpi, there was limited lesion development resulting from the inoculation and the edge of discs remained green. Cotyledon discs, inoculated or non-inoculated, derived from 15 random plants were pooled to form a biological replicate, with three independent replicates used for each treatment. A total of 18 samples were produced for subsequent RNA-seq. Samples from cotyledons inoculated with the *Lm* isolate 12CC329 (virulent), SC006 (avirulent) or water (mock) were designated respectively as locally compatible (COM-local, susceptible), incompatible (INC-local, resistant) interactions and mock inoculation (CK-local), while samples from the non-inoculated cotyledons of the same plants were named as COM-remote, INC-remote and CK-remote correspondingly. All samples were frozen in liquid nitrogen immediately after collection, and stored at − 80 °C.

### RNA isolation, library construction and sequencing

Total RNA was extracted using the RNeasy Plant Mini Kit (Qiagen, Toronto, ON) with on-column DNase digestion using a RNase-Free DNase Set (Qiagen) following manufacturer’s protocols. The RNA concentration was determined using a NanoDrop2000c spectrophotometer (Thermo Fisher Scientific, Wilmington, DE), and the RNA integrity was checked using a Bio-Rad Experion system (Bio-Rad Laboratories, Hercules, CA).

cDNA libraries were constructed using approximately 1 μg of total RNA per sample (replicate) with a TruSeq RNA Library Prep Kit v2 (Illumina, San Diego, CA) following manufacturer’s protocols. Briefly, poly (A) mRNA was first purified from total RNA using oligo (dT)-attached magnetic beads, chemically fragmented, and primed with random hexamer primer and SuperScript II reverse transcriptase (Life technologies, Burlington, ON) for first-strand cDNA synthesis. Subsequently, a second cDNA strand was synthesized as a replacement strand for the RNA to create double-stranded (ds) cDNA. The resulting overhanging ds cDNA fragments were converted to blunt ends and ligated to multiple sequencing adapters to prepare for hybridization. Finally, adaptor-ligated fragments were purified and enriched by PCR amplification to create final cDNA libraries. Following validation on the Bio-Rad Experion system and quantification via qRT-PCR, paired-end sequencing of the cDNA libraries was performed using MiSeq Reagent Kit v3 on a MiSeq platform (Illumina) to generate raw data with an average read length of 100 bp.

### Read mapping and gene-expression analysis

RNA-seq data were imported into CLC Genomics Workbench (v.8.5.1; Qiagen, Aarhus, Denmark) for read trimming, mapping and gene expression analysis. Raw reads were filtered by removing adaptor sequences, as well as low-quality reads (quality score < 0.05; ambiguous nucleotides > 2). The clean reads for each library (replicate) were subsequently mapped to the reference genome of *B. napus* (https://wwwdev.genoscope.cns.fr/brassicanapus/data/) using the v.5.0 gff3 annotation file with the following criteria: Mis-match cost was at 2, insertion cost at 3, deletion cost at 3, length fraction at 0.9, similarity fraction at 0.8 and maximum number of hits allowed at 1.

The value for the Reads Per Kilobase of exon model per Million mapped reads (RPKM) was calculated for each gene based on both the total number of reads mapped to the B. napus reference CDS database above (B. napus annotation v.5.0) and the corresponding gene length^55^ using the RNA-Seq Analysis in CLC. Only the genes with a absolute value of fold change ≥ 2 and a false discovery rate (FDR)^56^ ≤ 0.01 were deemed to be differentially expressed using the CLC Empirical Analysis tool, which is incorporated also in EdgeR Bioconductor^57^ and DESeq2 packages^58^. The variability among samples was examined using Principal Component Analysis (PCA) and the percent variation explained by each component calculated using the eigenvalue function in R FactoMineR package (v.2.3; http://factominer.free.fr)^59^.

### Gene ontology (GO) and Kyoto encyclopedia of genes and genomes (KEGG)

GO annotation and enrichment was performed using the Blast2Go PRO suite^59^. A sequence similarity search for all *B. napus* genes was carried out against the non-redundant protein database accessible at the National Center for Biotechnology Information (http://www.ncbi.nlm.nih.gov/) using BLASTX algorithm with an E-value threshold of 1e^−5^. All BLAST hits were then mapped to the GO database to retrieve GO terms that associated with each hit. All *B*. *napus* genes were searched subsequently against the InterPro (http://www.ebi.ac.uk/interpro/) and annotated by merging the results from Blast2GO and InterPro. GO terms that were significantly enriched for DEGs were identified by comparing with the whole genome background based on a false discovery rate (FDR) ≤ 0.01.

KEGG annotations were extracted from Ensemble Plant database using the R biomaRt package (v.2.44.1; https://bioconductor.org/packages/biomaRt/)^60^, and KEGG enrichment was performed using enricher function of the R clusterProfiler package (v.3.16.1; https://bioconductor.org/packages/clusterProfiler/)^61^. All expressed *B. napus* genes with annotation were used as a background, and KEGG pathways with an adjusted *p*-value < 0.05 were considered to be significantly enriched. Heat-maps of GO and KEGG enrichment were created using the R pheatmap package (v.1.0.12; https://CRAN.R-project.org/package=pheatmap).

### Gene co-expression analysis and heat map generation

To identify co-expression modules associated with Rlm1-mediated resistance, a co-expression network was generated using the weighted gene co-expression network analysis (WGCNA) package (v.1.69; https://CRAN.R-project.org/package=WGCNA)^62^ in R. Only the expressed genes with average RPKM values higher than 1 in any group of samples were retained and normalized in log2 scale using the rlog function in DESeq2 (v1.28.1; https://bioconductor.org/packages/DESeq2/)^58^ before further processing. The soft-thresholding power was determined using the pickSoftThreshold function. Hierarchical clustering was performed based on the topological overlap matrix (a concept defined for weighted networks) using the following parameters: Power was at 4, TOM-type unsigned, minModuleSize at 30 and maxModuleSize at 5000, to obtain maximum dynamic gene clusters. Initial clusters with similar expression profiles were merged at cutheight = 0.2. Heat-maps were generated using the R pheatmap package.

### Assessment of RNA-seq data quality with qRT-PCR

The reliability of RNA-seq data was validated by testing a subset of DEGs using quantitative real-time PCR (qRT-PCR) for the same RNA samples used in RNA-seq. The sequences of gene-specific PCR primers used in qRT-PCR are listed in Supplementary Table S7. The primers were designed using the Applied Biosystems Primer Express V3.0 (Life Technologies, Burlington, ON) and synthesized by Integrated DNA Technologies Inc. (Coralville, IA). The first strand of cDNA was synthesized using the Invitrogen SuperScript First-strand Synthesis system (Life technologies) from 1 μg of total RNA. qRT-PCR reactions were performed using the Power SYBR green master mix (Life technologies) on an ABI StepOne Plus Real-Time PCR system (Applied Biosystems, Foster City, CA) according to manufacturer’s instructions. The cycling conditions were as follows: samples were preheated at 95 °C for 10 min, followed by 40 cycles of 95 °C for 15 s, 58 °C for 30 s, and 60 °C for 30 s. Following amplification, melt-curve profiling was conducted to confirm the specificity of PCR amplification. Relative expression levels of target genes were normalized to the internal-control gene *ACTIN7* (GeneBank accession no. AF111812.1), and calculated according to the 2^–ΔΔCt^ method^63^. Three replicates were performed for each cDNA sample, with three samples (biological replicates) per treatment.

### Hormone treatments to validate pathways identified with RNA-seq analysis

All hormones were applied as a soil drench before cotyledon inoculation and as a foliar spray after the inoculation. In the soil-drench application, 10 ml of analytical-grade SA, methyl jasmonate (MeJA), ABA, auxin, gibberellin A3 (GA_3_) or kinetin (Sigma-Aldrich, St. Louis, MO) at the 1000 μM concentration was pipetted to the base of each canola seedling 24 h and 48 h, respectively, prior to inoculation. These soil-treated plants were also sprayed with the respective hormone solution in a misting bottle until run off at 24 h and 96 h after inoculation, respectively. Control plants were soil drenched and sprayed with equal volumes of water.

## Results

### Response of DH24288 to virulent and avirulent *Lm* isolates

**Figure 1:**
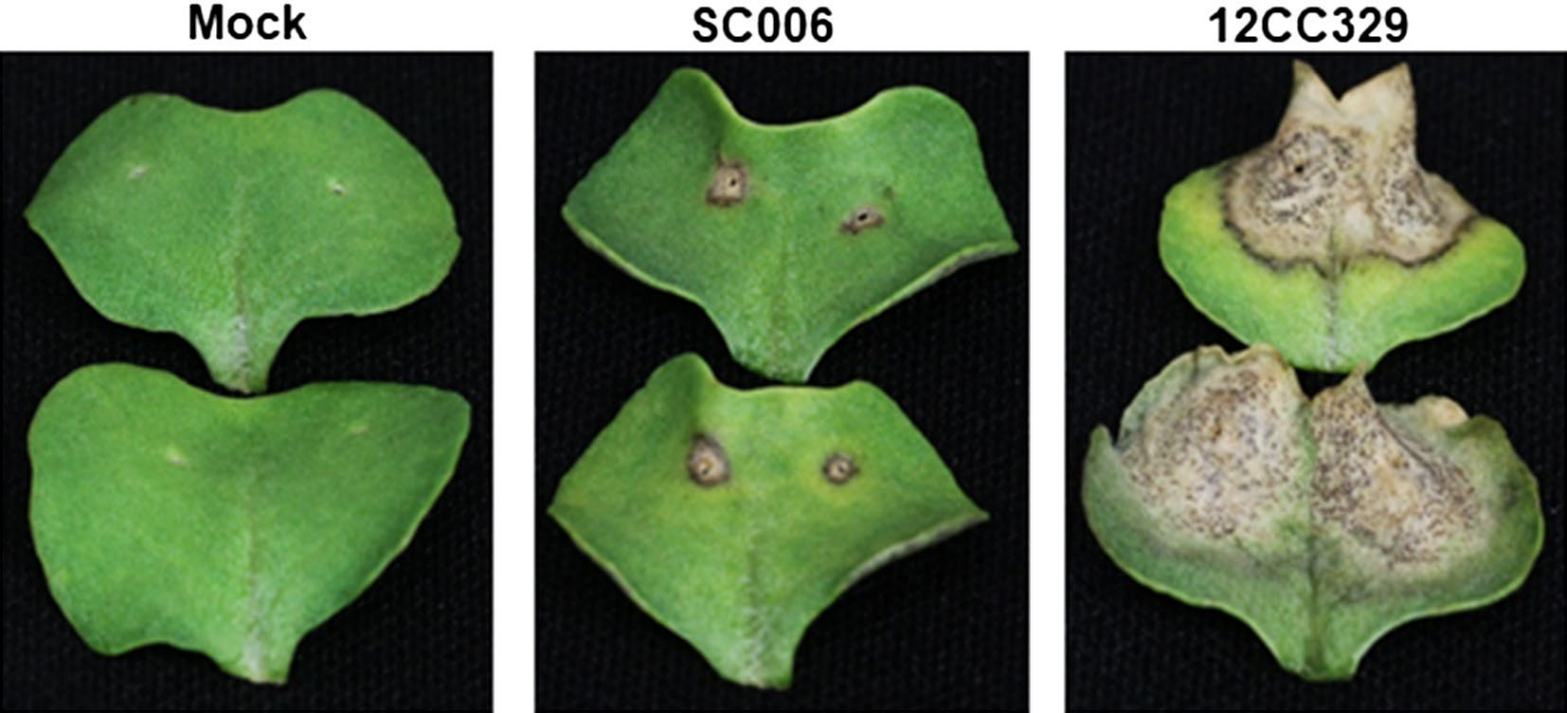
Phenotypic characterization of *B. napus* DH24288 (*Rlm1*) in response to *L. maculans* infection. Cotyledons of DH24288 were inoculated with water (Mock), *L. maculans* isolate SC006 (*AvrLm1*) or 12CC329 (*avrLm1*). Photos were taken at 14 dpi.

The inoculation of DH24288 (*Rlm1*) cotyledons with the isolate 12CC329 harbouring the virulent gene *avrLm1* caused a susceptible reaction, with large lesions spreading from the inoculation site at 14 days post inoculation (dpi) (Fig. 1). Pycnidia were clearly visible on these lesions. In contrast, the inoculation with the isolate SC006 carrying the avirulent gene *AvrLm1* resulted in a resistant reaction, with restricted lesion development. The small lesions tended to show sharp, dark margins.

### Global analysis of RNA-seq libraries

The gene expression profile of DH24288 was compared between compatible (susceptible) and incompatible (resistant) reactions based on RNA-seq of cDNA libraries constructed from inoculated and non-inoculated cotyledons. Approximately 243 million raw reads were generated from a total of 18 pooled samples (six treatments, each consisting of three biological replicates), with > 99.9% retrieved as clean reads after quality trimming. Of the clean reads, 52.3 to 70.9% were mapped uniquely to the *B. napus* reference genome, 4.1 to 5.9% aligned to multiple locations, and 23.5 to 43.6% failed to be mapped to the reference genome (Supplementary Table S1). The uniquely mapped reads corresponded to a total of 70,709 unigenes in the reference genome, with 58,700, 60,563 and 61,417 genes detected, respectively, in inoculated local tissues of susceptible (COM-local), resistant (INC-local) and mock (CK-local) reactions. Remote non-inoculated cotyledons were also sampled from inoculated plants for RNA-seq, with 60,754 (COM-remote), 60,179 (INC-remote) and 60,485 (CK-remote) unigenes identified, respectively, in these tissues remote to those with susceptible, resistant and mock-control reactions (Supplementary Table S1).

**Figure 2:**
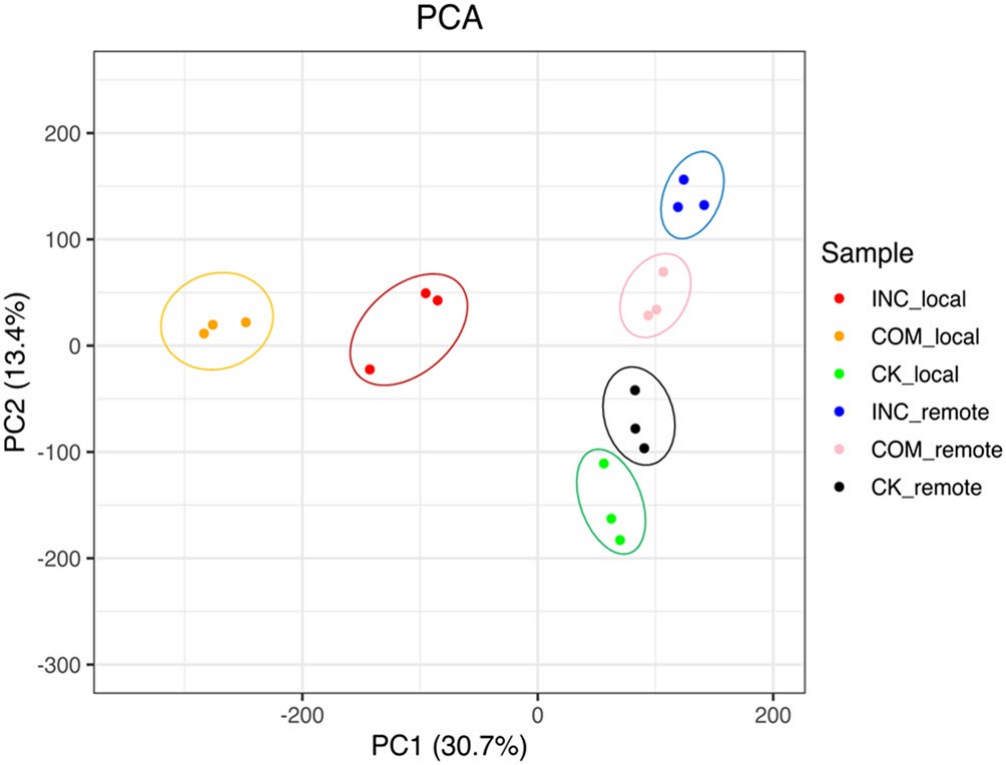
Principal component analysis (PCA) based on expressed genes across all samples. X-axis, PC1; Y-axis, PC2. The proportion of variance for each principal component is indicated in brackets following axis titles. Treatments are designated as compatible (COM, susceptible), incompatible (INC, resistant) and mock (CK) in local, inoculated (local) and remote, non-inoculated (remote) cotyledon tissues. Dots in the same color are replicates of same treatment.

Principal component analysis (PCA) was used to examine the characteristics of transcriptomes based on gene identity and expression levels (Fig. 2). Biological replicates were well clustered for each treatment and all treatments were separated from one another, indicating a good level of reproducibility of the RNA-seq data. Moreover, samples from both COM-local and INC-local showed clear separation from those of CK-local, indicating changes in transcriptome responding to both virulent and avirulent *Lm* isolates. In contrast, less variation was observed among COM-remote, INC-remote, CK-remote and CK-local samples, which may indicate that no dramatic response was induced by *Lm* at remote locations.

### DEGs in response to *Lm* infection

**Figure 3:**
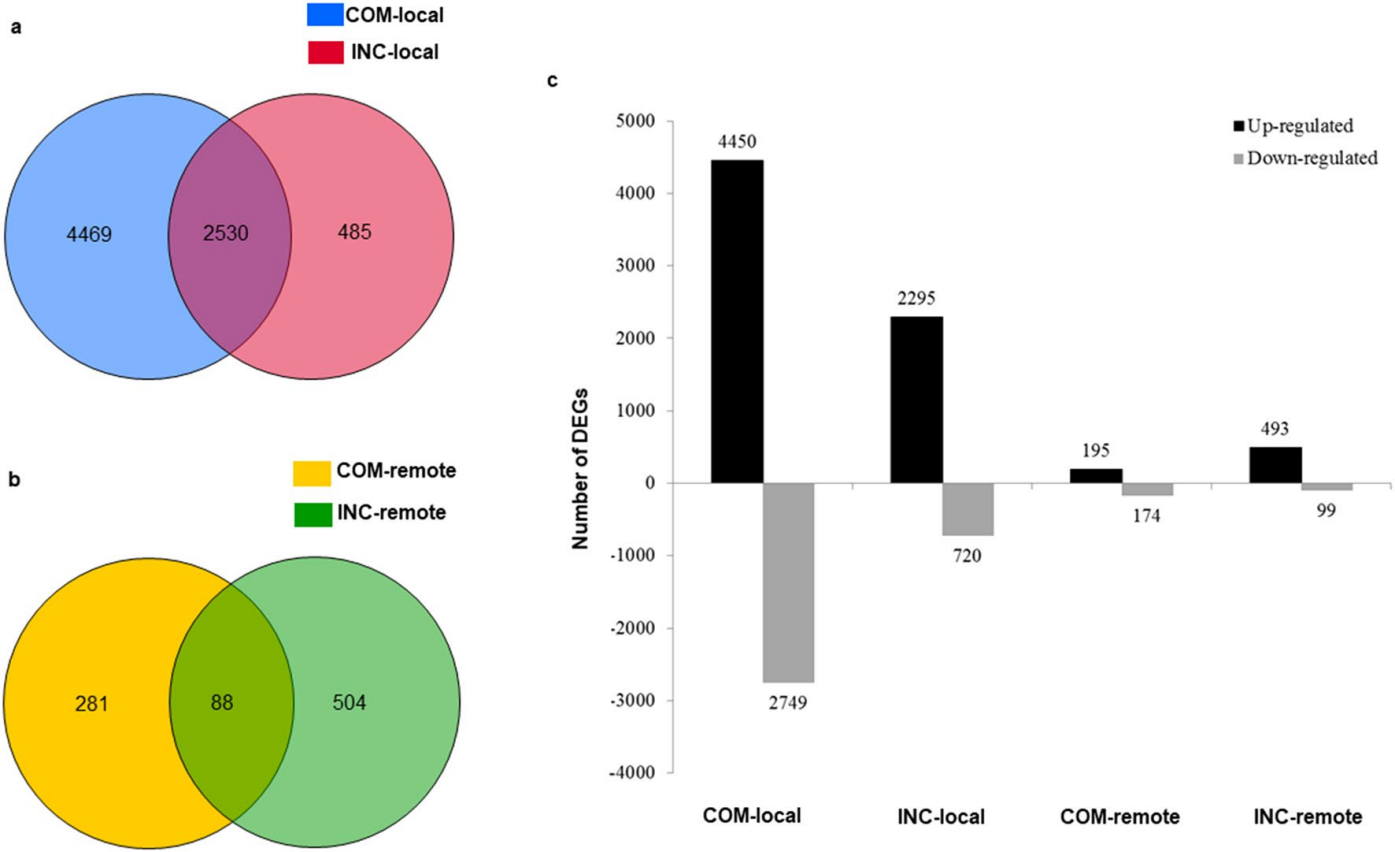
DEGs identified in the local cotyledon tissue of DH24288 inoculated with a virulent or avirulent *Lm* isolate, and in remote (non-inoculated) cotyledons at 7 dpi. (**a**) DEGs between local samples of susceptible (COM-local) and resistant (INC-local) reactions. (**b**) DEGs between remote cotyledon samples, COM-remote and INC-remote, on plants with locally susceptible and resistant reactions, respectively. (**c**) Up-regulated and down-regulated DGEs in each of the samples.

The level of gene expression was assessed against the mock control for DEG identification, with the false discovery rate (FDR) ≤ 0.01 and absolute value of fold change ≥ 2 applied as the cutoffs. Venn diagrams showed the distribution of DEGs between samples (Fig. 3). In inoculated local tissues, a total of 6999 and 3015 genes were differentially expressed in susceptible (COM-local) and resistant (INC-local) samples respectively, among which 4469 genes were uniquely identified in COM-local, whereas 485 were specifically expressed in INC-local (Fig. 3a). Though the host showed more pronounced responses to the virulent isolate, the proportion of the up-regulated DEGs in the resistant reaction (2295 DEGs, 76.1%) was higher than that in the susceptible reaction (4250 DEGs, 60.7%) (Fig. 3c, Supplementary Table S2). As for systemic responses in the non-inoculated cotyledons of the same plants, much fewer DEGs were detected. The numbers of up-regulated DEGs were 195 and 493, and down-regulated DEGs were 174 and 99 in remote susceptible (COM-remote) and resistant (INC-remote) samples, respectively (Fig. 3c, Supplementary Table S2). Only a small number of DEGs (88) overlapped between the two remote treatments (Fig. 3b), indicating the infection by virulent and avirulent *Lm* isolates *r*esulted in different host responses at locations remote from the inoculation site.

### GO classification and enrichment analyses

DEGs were further annotated and classified using GO terms for a better understanding of relevant biological functions. The majority of DEGs from inoculated local samples (94.1% for COM-local, and 93.5% for INC-local) had known functions; based on the biological process, molecular function and cellular component, those from COM-local displayed significant enrichment in 20, 5 and 9 subcategories while those from INC-local enriched the DGEs in 14, 5 and 8 subcategories, respectively. Similarly, Blast2GO yielded annotated sequences for 93.5% and 95.4% of DEGs, respectively, in COM-remote and INC-remote samples. DEGs in COM-remote and INC-remote samples were classified respectively into 34 and 35 functional groups covering 3 categories. The detailed GO classification of DEGs is shown in Supplementary Fig. S1 and Table S3.

**Figure 4:**
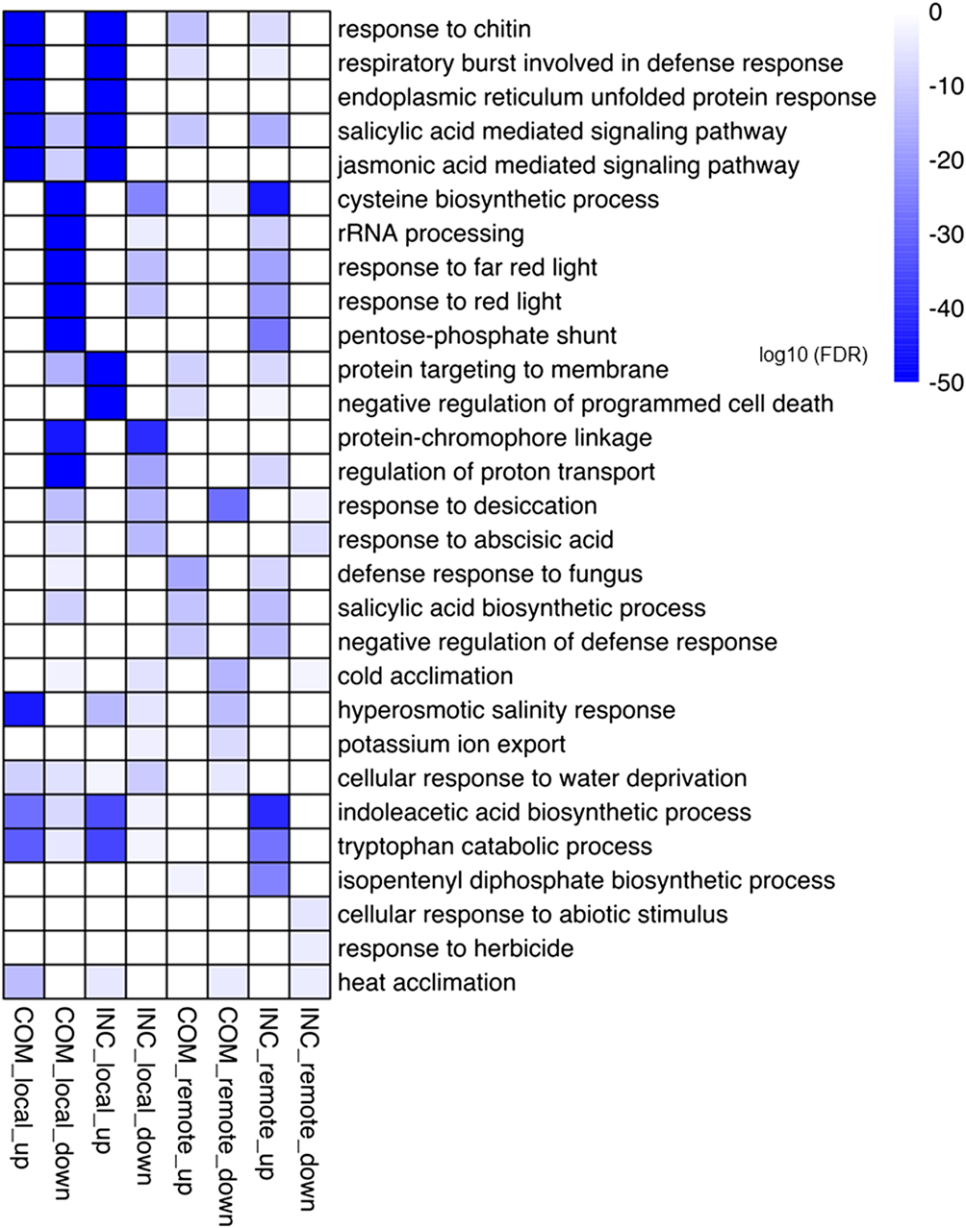
Top five GO terms enriched for the up-regulated and down-regulated DEGs in COM-local, INC-local, COM-remote and INC-remote cotyledon samples. The significance of FDR is associated with colors, from white (high) to blue (low), as defined by the inset.

To further identify the enrichment associated with DEGs in specific biological processes, GO enrichment was analyzed using Fisher’s Exact Test of Blast2GO data. At FDR < 0.01, a total of 305 and 178 enriched GO terms were identified, respectively, for COM-local and INC-local, with 160 and 129 of them being associated with up-regulated DEGs, and the rest with down-regulated DEGs (Supplementary Table S4). The top five enriched GO terms associated with each group of samples are shown in Fig. 4; many defense-related biological processes, including “response to chitin”, “respiratory burst involved in defense response”, “endoplasmic reticulum unfolded protein response”, “salicylic acid (SA)-mediated signaling pathway” and “jasmonic acid (JA)-mediated signaling pathway” were commonly enriched for up-regulated DEGs in both COM-local and INC-local samples. Intriguingly, signal transduction pathways for the two phytohormones, i.e. SA- and JA-mediated signaling pathways, were also enriched among down-regulated DEGs in COM-local, but not in INC-local samples. These results may indicate that similar defense responses are triggered for susceptible and resistant reactions involving *avrLm1* (virulent) and *AvrLm1* (avirulent) against the *R* gene *Rlm1*, but the aptitude of the response regulated by these signaling pathways may vary between the interactions.

“Protein targeting to membrane” and “negative regulation of programmed cell death” were exclusively overrepresented among up-regulated DEGs in INC-local samples. In contrast, more biological processes related to photosynthesis and growth/development/morphogenesis were enriched among down-regulated DEGs in COM-local than INC-local samples, including “chlorophyll biosynthetic process”, “chloroplast relocation”, “photosynthetic electron transport in photosystem I”, “photosynthesis, light harvesting in photosystem I”, “chlorophyll catabolic process”, “photosystem II stabilization”, “photosystem II repair”, “photosynthetic electron transport in photosystem II” and “photosystem I stabilization”, “root-hair elongation”, “cell tip growth”, “multidimensional cell growth”, “regulation of meristem growth”, “cuticle development”, “leaf morphogenesis” and “stomatal complex morphogenesis” (Supplementary Table S4). It appears that successful colonization by *Lm* also results in a more pronounced decline in photosynthesis and cell growth/development in the infected locality.

GO enrichment analysis retrieved 59 and 86 GO terms, respectively, in COM-remote and INC-remote samples (Supplementary Table S4). General defense responses were similarly overrepresented among up-regulated DEGs in both COM-remote and INC-remote samples, including “response to chitin”, “respiratory burst involved in defense response” and “defense response to fungus” (Fig. 4). Notably, both “SA biosynthetic process” and “SA mediated signaling pathway” were also elevated in these two remote samples (Fig. 4), implying potential induction of SAR. Moreover, “cysteine biosynthetic process”, “tryptophan catabolic process” and “pentose-phosphate shunt” were exclusively enriched for up-regulated DEGs in INC-remote samples (Fig. 4). These results showed that infection by virulent or avirulent *Lm* isolate also led to changes in the biosynthesis and metabolism of remote tissues.

### KEGG pathway enrichment analysis

**Figure 5:**
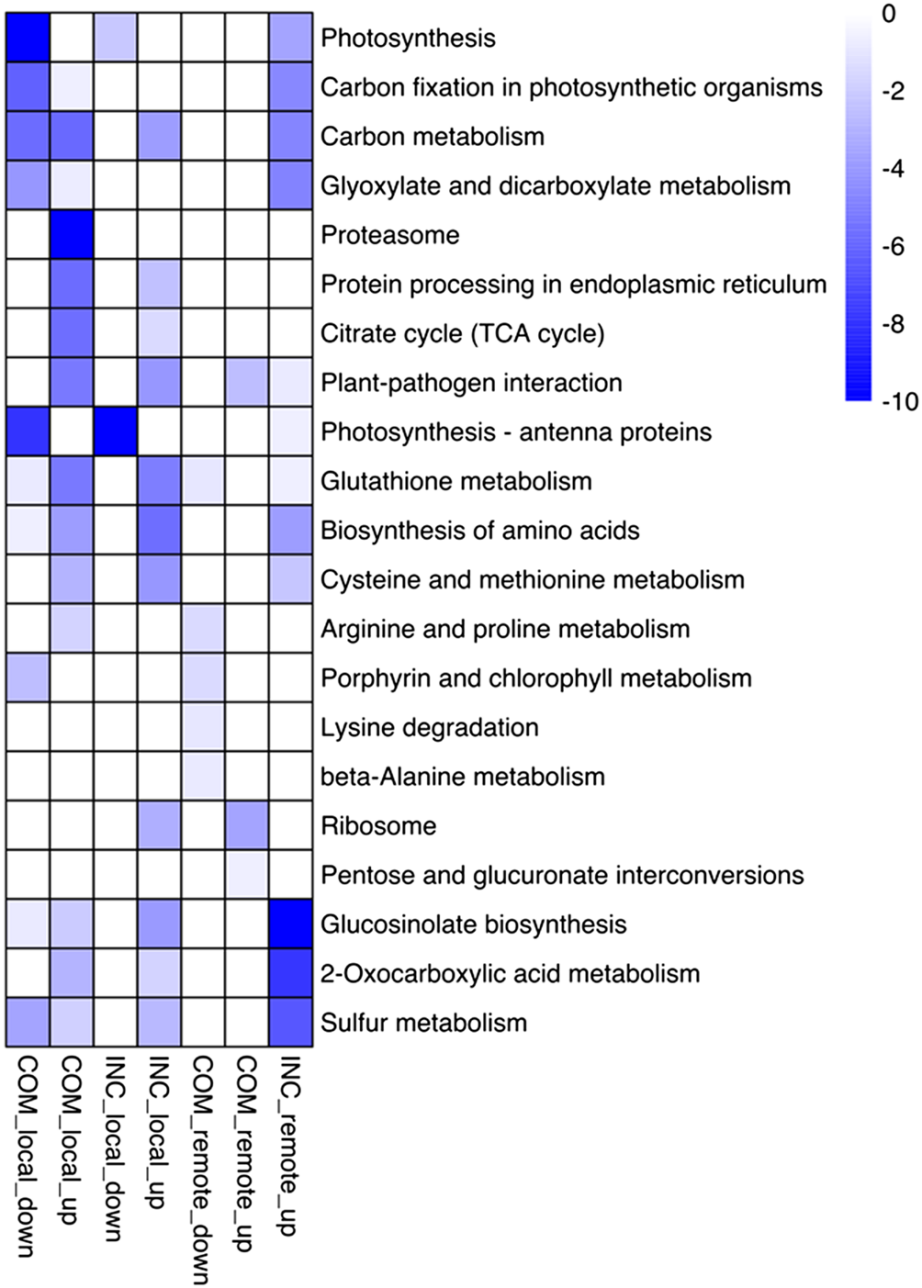
KEGG pathways enriched for up- and down-regulated DEGs in COM-local, INC-local, COM-remote and INC-remote treatments. Pathways with a significant adjusted *p*-value (< 0.05) are shown in association with colors from white (high) to blue (low), as defined in the inset.

To explore the main metabolic pathways in which DEGs are involved, KEGG pathway enrichment analysis was conducted at adjusted *p* < 0.05 (Fig. 5). In total, 21 KEGG pathways were identified, among which “carbon metabolism”, “protein processing in endoplasmic reticulum”, “TCA cycle”, “plant-pathogen interaction”, “glutathione metabolism”, “biosynthesis of amino acids”, “cysteine and methionine metabolism”, “2-Oxocarboxylic acid metabolism”, “glucosinolate biosynthesis” and “sulfur metabolism” represented the most common pathways enriched among up-regulated DEGs in both COM-local and INC-local samples. “Proteasome” was associated with up-regulated DEGs in COM-local only, while “ribosome” was associated with up-regulated DEGs in INC-local only. For down-regulated DEGs, “photosynthesis” and “photosynthesis–antenna proteins” were enriched in both COM-local and INC-local samples. “Carbon fixation in photosynthetic organisms” was identified only in COM-local, which was consistent with GO enrichment results of suppressed photosynthesis in COM-local samples.

For remote samples, “plant-pathogen interaction” was the only pathway enriched among up-regulated DEGs in both COM-remote and INC-remote (Fig. 5). Overall, more metabolic pathways were enriched for up-regulated DEGs in INC-remote relative to COM-remote, including “glucosinolate biosynthesis”, “2-oxocarboxylic acid metabolism”, “sulfur metabolism”, “glyoxylate and dicarboxylate metabolism”, “carbon metabolism”, “biosynthesis of amino acids”, “cysteine and methionine metabolism” and “glutathione metabolism”, which was also consistent with GO enrichment results. The three photosynthesis-related pathways, i.e. “photosynthesis”, “photosynthesis—antenna proteins” and “carbon fixation in photosynthetic organisms”, were also exclusively enriched among up-regulated DEGs in INC-remote, indicating altered metabolism leading to elevated photosynthesis in these remote tissues. For down-regulated DEGs, “porphyrin and chlorophyll metabolism”, “arginine and proline metabolism”, “lysine degradation”, “glutathione metabolism” and “beta-Alanine metabolism” were enriched in COM-remote tissues, whereas no pathway was enriched for down-regulated DEGs in INC-remote (Fig. 5).

### Core genes involved in *Rlm1*-mediated defense network against avirulent *Lm*

Plant defense response can be triggered following the perception and recognition of a pathogen, with subsequent signal transduction and transcriptional reprogramming which activate additional resistance-related genes. GO-term annotation for DEGs from inoculated local samples may allow us to focus on the core genes involved in the *Rlm1*-mediated defense network at different levels.

#### Recognition of *Lm* infection by receptor-like protein kinases R proteins

**Figure 6:**
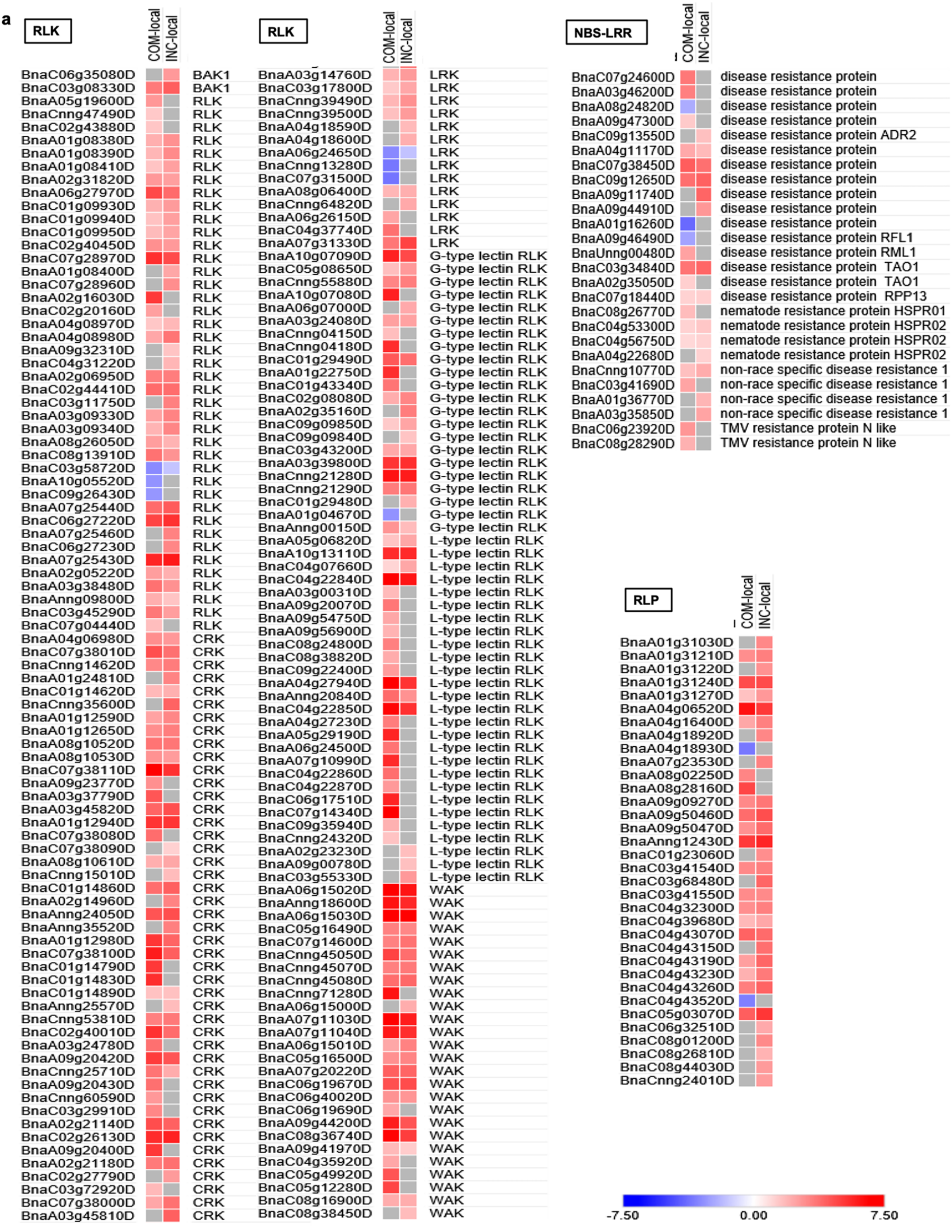

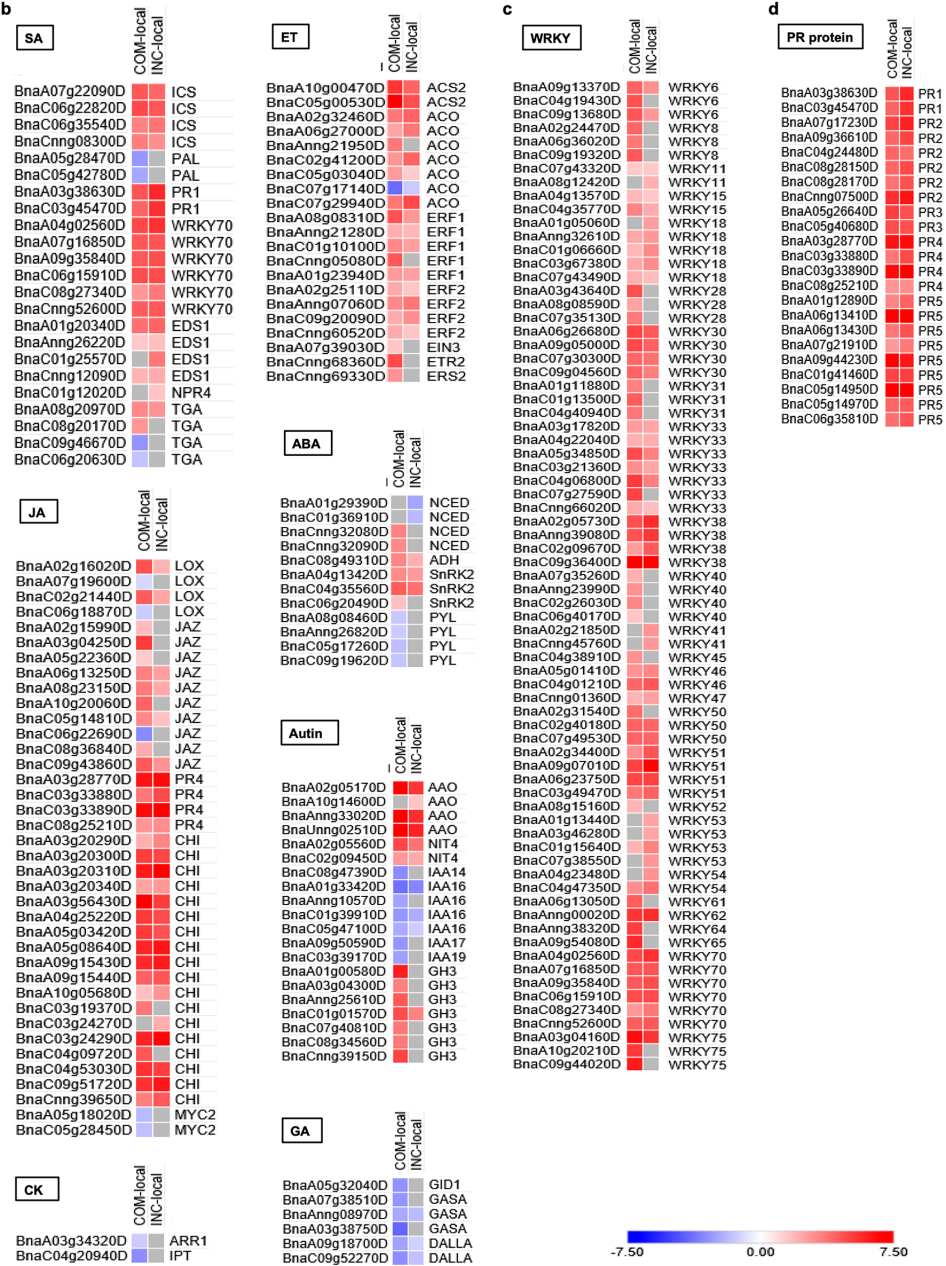
Heat map illustration of the core genes involved in the defense network against *Lm* infection. (**a**) DEGs encoding RLKs, NBS-LRRs and RLPs. (**b**) DEGs involved in phytohormone pathways. (**c**) DEGs encoding WRKY TFs. (**d**) DEGs encoding PR proteins. The colour bar represents values of log_2_-fold changes ranging from − 7.5 (blue) to 7.5 (red).

The perception of pathogen usually depends on non-specific recognition of conserved microbial structures via membrane-localized receptor-like protein kinases (RLKs), or specific recognition of pathogen effectors by nucleotide-binding site leucine-rich repeat (NBS-LRR) proteins. In this study, 177 genes encoding RLKs were differentially expressed in response to *Lm* infection, with 148 in COM-local and 126 in INC-local samples (Fig. 6a, Supplementary Table S5). Of these DEGs, 29 were specifically induced and 51 were more significantly up-regulated in INC-local, relative to those in COM-local tissues. Several types of RLKs, including cysteine-rich RLKs (CRKs), leucine-rich repeat RLKs (LRKs), G-type lectin RLKs, L-type lectin RLKs and wall-associated RLKs (WAKs), were among the DEGs specifically or more significantly induced in INC-local samples.

A total of 26 DEGs were annotated as NBS-LRR genes, with 20 of them found in COM-local and 14 in INC-local samples (Fig. 6a, Supplementary Table S5). Notably 12 of these DEGs, including those homologous to the Arabidopsis *R* genes *ADR2* (*BnaC09g13550D*), *RPP13* (*BnaC07g18440D*) and *HSPRO2* (*BnaC04g53300D*, *BnaC04g56750D*, *BnaA04g22680D*) were exclusively induced or more significantly up-regulated in INC-local samples, while three of the DEGs, including the homologs of Arabidopsis *R* gene *RFL1* (*BnaA09g46490D*), were exclusively down-regulated in COM-local samples. None of the above NBS-LRR-coding DEGs, however, were located within the *Rlm1-*mapping region on chromosome A7. Since the blackleg-resistance genes *LepR3* and *Rlm2* have been cloned and proved to encode RLPs^32,33^, the expression of putative RLP-coding DEGs identified in this study were further investigated. In total 34 of the DEGs were annotated as RLPs, with 28 of them up-regulated exclusively or more significantly in INC-local, and two down-regulated exclusively in COM-local samples (Fig. 6a, Supplementary Table S5**).** The DEG *BnaA07g23530D*, which was induced specifically in INC-local samples, was located within the mapped region for *Rlm1*^64^ and therefore considered a strong candidate responsible for *Rlm1*-mediated resistance.

#### Phytohormone signaling in response to *Lm* inoculation

Several phytohormone pathways were indicated in the GO enrichment, including SA, JA, ethylene (ET), abscisic acid (ABA), auxin, gibberellic acid (GA) and cytokinin (CK), in response to *Lm* inoculation (Supplementary Table S4). Heat-map analysis based on the expression level of genes involved in these pathways supported a modulation pattern for all these hormones, except ET, between COM-local and INC-local samples, i.e. the biosynthesis and signaling of SA and JA were induced more significantly in INC-local, the biosynthesis of ABA and auxin were induced more strongly in COM-local, and the signaling of GA and CK were repressed more significantly also in COM-local samples (Fig. 6b, Supplementary Table S5). For instance, genes encoding *ICS*, which is involved in the isochorismate synthase pathway for SA biosynthesis, were induced in both COM-local and INC-local samples, but genes encoding *PAL*, which are involved in the phenylalanine ammonia-lyase pathway for SA biosynthesis, were repressed only in COM-local samples. All copies of *PR1*, *WRKY70*, *EDS1* and *NPR4*, which are involved in SA signaling, were exclusively or more significantly induced in INC-local, and two copies of *TGA* were exclusively down-regulated in COM-local samples.

*WRKY70*, *EDS1*, *NPR4* and *TGA* have been proven to up-regulate SA pathways, and *PR1* is an SA-responsive defense gene^40,65^. All copies of *LOX*, which are involved in JA biosynthesis, were up-regulated in INC-local, while two of the copies were down-regulated in COM-local samples. As for JA signaling, most copies of *PR4* and *CHI* were more significantly induced in INC-local, while all copies of *MYC2* were exclusively down-regulated and most copies of *JAZ* more significantly up-regulated in COM-local samples. *PR4* and *CHI* are known as JA-responsive defense genes involved in the ERF branch of JA signaling, while *MYC2* and *JAZ* induces and represses the MYC branch of JA signaling^40,66^, respectively. Additionally, the major genes related to ABA (*NCED*, *ADH*) and auxin (*AAO*, *NIT4*) biosynthesis were more significantly induced in COM-local, while the genes encoding the core component of GA (*GID1*, *GASA*, *DELLA*) and CK (*ARR1*, IPT) signaling were exclusively or more strongly repressed in COM-local samples.

#### *WRKY* TFs responsive to *Lm* inoculation

WRKY proteins comprise the best-characterized family of TFs involved in controlling transcriptional reprogramming to mediate cellular responses to pathogen attacks^43^. A total of 73 DEGs encoding 26 members of the WRKY gene family, e.g. *WRKY6*, *8*, *11*, *15*, *18*, *28*, *30*, *31*, *33*, *38*, *40*, *41*, *45*, *46*, *47*, *50*, *51*, *52*, *53*, *54*, *61*, *62*, *64*, *65*, *70* and *75*, were identified, with 65 from COM-local and 26 from INC-local samples (Fig. 6c, Supplementary Table S5). Of these, *WRKY8*, *28*, *31*, *40*, *45*, *52*, *61*, *64* and *65* were identified only in COM-local, and *WRKY41* was specifically induced in INC-local samples. As for the *WRKY* genes identified in both COM-local and INC-local samples, the expression level and/or copy number was higher for WRKY11, 18, 38, 46, 47, 51, 53, 54, 62 and 70 in INC-local than those in COM-local samples. Notably, most of them have been reported/implicated for positive regulation of plant defense responses against different pathogens^48,67–71^.

#### Pathogenesis-related (PR) proteins in response to *Lm* infection

The production and accumulation of PR proteins represent an important change in host protein composition in response to invading pathogens, and have been shown to correlate with plant disease resistance^72,73^. In total, 23 genes encoding *PR* proteins were up-regulated in both COM-local and INC-local samples, including multiple copies of *PR1*, *2*, *3*, *4* and *5* (Fig. 6d, Supplementary Table S5). The PR1 family are still unknown proteins, the PR2 family belongs to β-1,3-glucanase, PR3 and PR4 groups are comprised of chitinases, and the PR5 group is a thaumatin-like protein. All of these PR proteins can restrict the growth of fungi^73^. Heat-map analysis showed that all copies of *PR1* and *PR4*, as well as most copies of *PR2* and *PR5*, were up-regulated more significantly in INC-local, while all copies of *PR3* were induced more significantly in COM-local samples (Fig. 6d, Supplementary Table S5).

### Gene co-expression modules regulating *Rlm1*-mediated resistance

**Figure 7:**
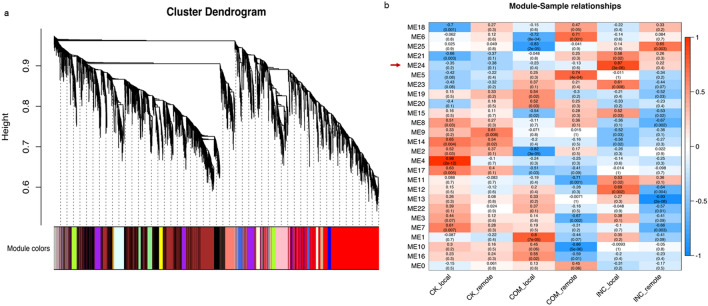
Weighted gene co-expression network analysis (WGCNA) across all treatments. (**a**) Hierarchical clustering tree (dendrogram) of DEGs with dissimilarity based on the topological overlap. Each color represents one specific co-expression module, the branches above represent genes, and the height (y-axis) indicates the level of correlation. (**b**) Correlation relationship between modules and treatments. Each row corresponds to a module eigengene, and each column corresponds to a treatment. Each cell contains a corresponding correlation (top) and *p*-value (bottom). The color scheme, from red through white to blue, indicates the level of correlation, from high to low. The module eigengene (ME24; *r* > 0.85, *p* < 0.001) closely related to INC-local is indicated by a red arrow.

To identify co-expression modules associated with the *Rlm1*-mediated defense response, a total of 38,020 genes with average RPKM values > 1 in any group of samples were examined via weighted gene co-expression network analysis (WGCNA; Supplementary Table S6). The soft-thresholding power, which affects the scale independence and mean connectivity of the network, was adopted in the network topology. Following screening, a soft-threshold of 4, which was the lowest power with the scale-free topology fit index reaching 0.90, was chosen to generate a hierarchical clustering tree (dendrogram). As shown in Fig. 7a, 25 color modules which composed of genes with similar expression patterns were identified. The size of these modules depends on the number of genes involved, ranging from 35 to 18,122 (Supplementary Table S6).

The relationship between each module and treatment was evaluated by correlating the eigengenes of each module with each group of samples. One module, named ME24, exhibited significant correlation (r = 0.87; *p* = 3e−6) with INC-local samples (Fig. 7b), implying its involvement in *Rlm1*-mediated resistance. A total of 61 genes were clustered in this module, of which 26 were also DEGs identified in INC-local samples (Supplementary Table S6). Notably, the DEG *BnaA10g05680D* is a *CHI*-encoding gene involved in JA signalling. Moreover, four of the DEGs (*BnaA02g23230D*, *BnaA04g18600D*, *BnaA06g07000D* and *BnaC04g31220D*) encode RLKs and another four (*BnaC01g06660D*, *BnaCnng45760D*, *BnaA01g13440D* and *BnaC07g38550D*) encode WRKY proteins WRKY18, 41 or 53.

### Validation of RNA-seq data

**Figure 8:**
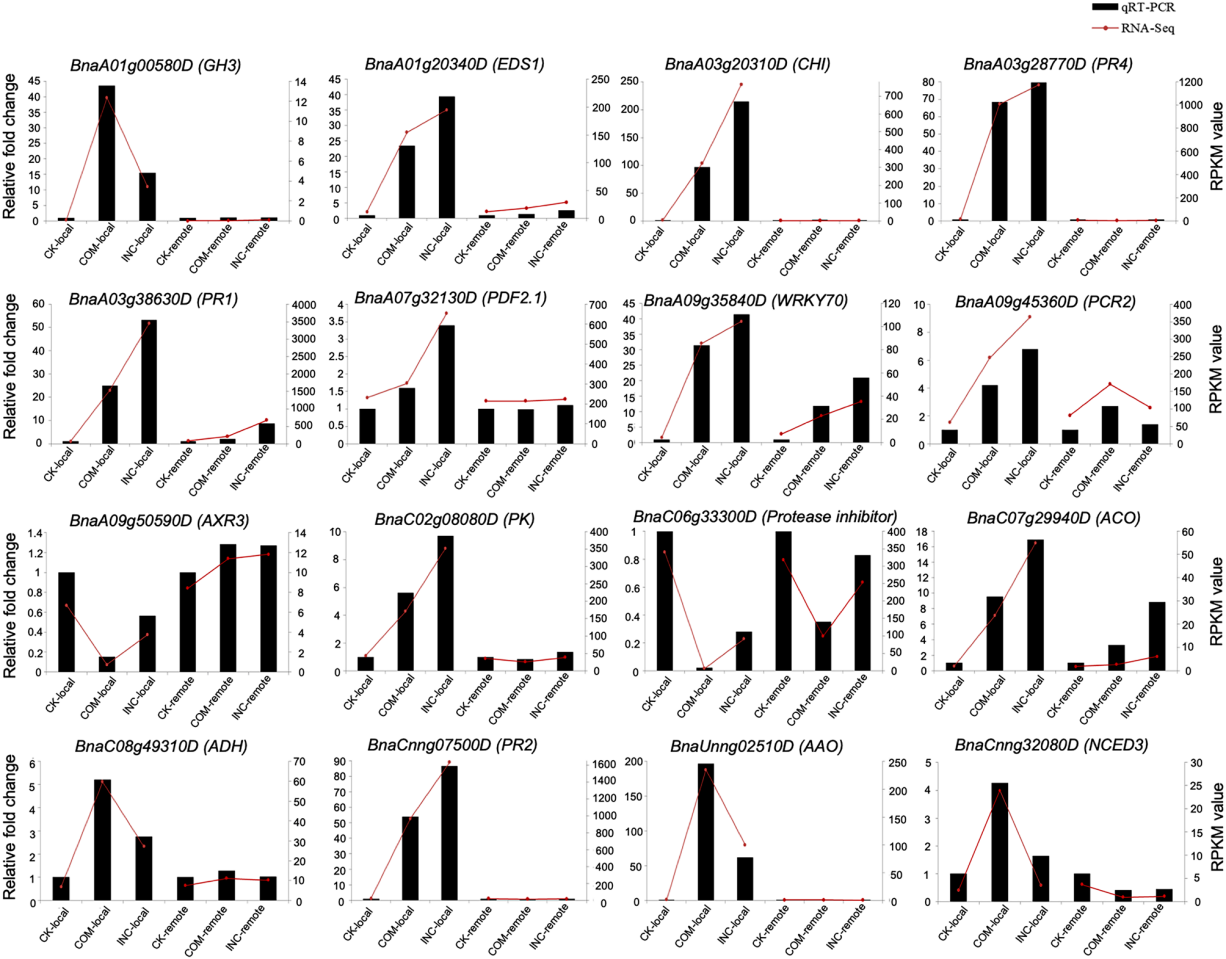
Expression profile of selected DEGs in RNA-seq and qRT-PCR analyses. Red lines indicate the transcript abundance of RNA-seq (right y-axis) based on RPKM, while black bars represent the relative expression level against the average value of mock (CK-local or CK-remote) in qRT-PCR.

To determine the overall reliability of RNA-seq results, 16 DEGs with a range of expression levels and patterns were tested with qRT-PCR, including those involved in pathogen recognition, transcriptional regulation, hormone regulation and defense responses based on the functional annotation. These DEGs showed the expression similar to those in the RNA-seq analysis across treatments, validating the RNA-seq analysis conducted (Fig. 8).

Exogenous applications of SA to DH24288, pre and post inoculation of cotyledon using the virulent (*avrLm1*) isolate, reduced the infection severity substantially relative to water controls, while the JA treatments resulted in slightly smaller effect (Supplementary Fig. S3). Since the heat map also indicated the activation of ABA and auxin biosynthesis, as well as the suppression in GA and CK signaling in the susceptible reaction (Fig. 6b, Supplementary Table S5), ABA, auxin, GA and cytokinin were also tested in exogenous applications but showed little effect on symptom development (data not shown).

Pre-inoculation of one of the cotyledons with a virulent (12CC329) or avirulent (SC006) *Lm* isolate, followed by challenging the other cotyledon with the virulent isolate three days later failed to reduce the symptom caused by the challenge inoculation substantially, relative to the mock pre-inoculation (Supplementary Fig. S4).

## Discussion

Despite extensive studies on genetic resistance against blackleg of canola, resistance mechanisms have yet to be well characterized. In this study, the high-throughput RNA sequencing was used to decipher key interactions between a DH *Bn* line carrying *Rlm1* and *Lm* isolates carrying or not carrying the corresponding *Avr* gene *AvrLm1*. The identification of DEGs, as well as functional characterization, help capture molecular signatures during the *Bn–Lm* interaction and potentially shed light on the modes of action for *Rlm1-*mediated resistance. The information on the pathological and transcriptional changes in uninfected tissues remote from the inoculated cotyledon may help determine whether the *Rlm1*-based resistance can be systemic.

### Both PTI and ETI are involved in host defense responses mediated by *Rlm1*

The plant immunity can generally be divided into PTI and ETI^34^, with PTI often being the first step of active defense initiated by the recognition of PAMPs through PPRs. Most PRRs are RLKs with an extracellular receptor domain, a transmembrane domain, and typically a cytoplasmic kinase domain^74^. In this study, 177 genes encoding RLKs were differentially expressed in response to *Lm* infection, with 148 in COM-local and 126 in INC-local samples (Fig. 6a, Supplementary Table S5). Identification of RLK-encoding DEGs in both COM-local (susceptible) and INC-local (resistant) might suggest PTI is activated by both virulent and avirulent isolates. It is noteworthy that four of the RLK-encoding genes induced in INC-local were also members in gene module that correlated with the resistant reaction (Supplementary Table S6), implying their potential role against the infection by the avirulent *Lm* isolate at the PTI stage.

ETI is a stronger defense response activated by host recognition of specific effectors from a pathogen^34^, mediated generally by intracellular NBS-LRR proteins. However, pathogens like *Lm* generally deliver their effectors into apoplast rather than host cell; the recognition of apoplastic effectors in intercellular spaces thus relies on cell-surface RLPs that are integral plant membrane proteins containing an extracellular LRR domain and a short cytoplasmic tail without a signaling motif^74^. In fact, RLPs are involved in resistance to blackleg by *LepR3* and *Rlm2*^32,33^. In the present study, 34 DEGs were annotated as RLPs (Fig. 6a, Supplementary Table S5), and among them, *BnaA07g23530D* was significantly up-regulated in INC-local and located within the region mapped for *Rlm1*^64^. It is therefore considered a candidate gene for *Rlm1*-mediated resistance. The expression pattern was investigated for genes in the *Rlm1-*mapped region using heat-map analysis (Supplementary Fig. S2). Of 61 expressed genes in this region, 34 were up-regulated and 26 down-regulated consistently in both COM-local and INC-local samples. One gene without annotation, i.e. *BnaA07g21170D*, was up-regulated in COM-local but down-regulated in INC-local samples. Three genes encoding RLKs, i.e. *BnaA07g25430D*, *BnaA07g25440D* and *BnaA07g25460D*, were more induced in INC-local than those in COM-local and may merit further investigation for being potentially involved in *Rlm1*-mediated blackleg resistance.

### Both SA and JA may be required for the activation of *Rlm1*-mediated resistance

Phytohormones, including SA, JA, ET and ABA, are essential regulators of host defense against various pathogens^40,75–77^. Additionally, auxin, GA and CK have also been implicated in the modulation of plant-pathogen interactions^77–83^. In this study, several phytohormone pathways were activated by *Lm* infection based on the GO analysis, especially the significant enrichment of SA- and JA-related biological processes with both up- and down-regulated DEGs for COM–local, but only up-regulated DEGs for INC-local (Fig. 4, Supplementary Table S4). Heat-map analysis based on the expression level of major genes involved in these pathways revealed that all hormone pathways, except ET, displayed different modulation patterns in susceptible (COM–local) and resistant (INC-local) reactions. Noticeably, both biosynthesis and signaling of SA and JA were induced more significantly in the resistant reaction (Fig. 6b, Supplementary Table S5). WGCNA analysis also showed that *BnaA10g05680D,* a JA-responsive defense gene identified as a DEG in INC-local samples, was clustered in the gene module closely correlated with the resistant reaction (Fig. 6b, Supplementary Table S6). Taken together, the results indicate SA and JA biosynthesis and signaling are possibly involved in *Rlm1*-mediated blackleg resistance.

It appears that SA- and JA-related defense pathways can be triggered by *Lm* in both susceptible and resistant reactions, but a threshold level of signaling may be required to activate *Rlm1-*mediated resistance. Virulence proteins from *avrLm1* may limit the expression of core genes required to propel these pathways to threshold levels. Becker et al.^51^ reported the importance of SA and JA pathways in a *Bn* line carrying the *R* gene *LepR1* interacting with a *Lm* isolate containing the cognate *Avr* effector *AvrLepR1*. However, in another study based on qPCR analyses, the recognition of *AvrLm1* by a *Bn* cultivar carrying *Rlm1* triggered greater expression of genes involved in SA and ET but not JA pathways^84^. The varied results between this and our study may be attributed to the limited numbers of genes that can be measured with qPCR, as opposed to DGEs based on RNA-seq and GO analysis. However, it was also noticed in the current study that SA induced stronger defense responses than JA based on exogenous application of these hormones (Supplementary Fig. S3), supporting a notion that SA may play a bigger role in *Rlm-1* mediated resistance.

Hormone crosstalk allows the flexibility of plants to adjust their inducible defense machineries in response to different pathogens^85^. However, SA and JA signaling may be mutually antagonistic in a range of pathosystems, as reviewed by Thaler et al.^86^. SA is commonly involved in resistance to biotrophic/hemibiotrophic pathogens, whereas JA is often associated with defense responses against necrotrophic pathogens^40^. Our results that both SA and JA pathways are activated in the resistance also indicate potential synergy between these two pathways. Similar results have been reported previously; Halim et al.^87^ found that Pep-13, a pathogen-associated molecular pattern from the hemibiotrophic pathogen *Phytophthora infestans* that acts as an elicitor, activated the defense responses of potato plants via both SA and JA pathways. Lemarié et al.^88^ also found that both SA and JA contributed to the resistance against clubroot caused by the biotrophic pathogen *Plasmodiophora brassicae* in Arabidopsis. These variably deployed mechanisms may represent positive and negative feedback loops that may allow plant responses tailoring to different pathogens^89^. *Lm* is a hemibiotrophic fungal pathogen with a transient biotrophic phase before the necrotrophic colonization that typically results in leaf lesions. At 7 dpi when samples were taken for RNA-seq, the fungus may still be transitioning from biotrophic to necrotrophic colonization, activating DEGs involved in both SA and JA pathways. Sampling in a time-course for RNA-seq may help show these changes more precisely.

### TFs and defense-related proteins

The enforcement of TFs network enables plants to mount successful immunity in a highly dynamic and temporal manner, and the plant-specific WRKY TFs comprise one of the largest families of regulatory proteins in this network^43,90^. In this study, a total of 73 WRKY genes were differentially modulated in response to *Lm* infection, and among them, *WRKY41* was induced specifically in INC-local (Fig. 6c, Supplementary Table S5). This is in line with the finding by Higashi et al.^68^, in which the expression of *WRKY41* was induced in Arabidopsis inoculated with an avirulent *Pseudomonas syringae* isolate but not with a virulent one. In our study, *WRKY11*, *18*, *38*, *46*, *47*, *51*, *53*, *54*, *62*, and *70* were more significantly up-regulated in INC-local than in COM-local (Fig. 6c, Supplementary Table S5); these TFs have also been found to play a positive role in plant defense responses^48,67–71^. For instance, the overexpression of *WRKY11* enhanced the resistance of rice plants to *Xanthomonas oryzae*, as well as the tolerance to drought stress^71^. Coordination between *WRKY46*, *WRKY53* and *WRKY70* contributed to basal resistance against *P. syringae* in Arabidopsis^69^. Notably, WGCNA analysis revealed the significant correlation of four genes encoding WRKY18*,* 41 and 53 (*BnaC01g06660D*, *BnaCnng45760D*, *BnaA01g13440D* and *BnaC07g38550D*) with the resistance (Supplementary Table S6), indicating their role as the key node of *Rlm1*-mediated regulatory network.

One of the most important and effective plant defense mechanisms is the activation of defense-related genes which result in the production and accumulation of PR proteins^72^. To date 17 groups of PR proteins (*PR1* to *PR17*) have been identified, five (*PR1* to *PR 5*) of which were significantly induced in the resistant reaction of this study. All *PR1* and *PR4* copies, as well as most of the *PR2* and *PR5* copies, were strongly up-regulated in resistant samples, while all *PR3* copies were more induced in susceptible samples (Fig. 6d, Supplementary Table S5). The function of the *PR1* family is still not well understood, although it has been thought to be associated with cell-wall thickening against apoplastic pathogens in SAR^72,91^. The β-1,3-glucanase *PR2* and chitinase *PR3/PR4* affect the cell wall of fungal pathogens and promote the release of cell-wall derived fragments that can act as resistance elicitors^73,92^. The *PR5* thaumatin-like proteins are similar to the bifunctional α-amylase proteinase inhibitor of maize, which may induce plant resistance via inhibiting pathogen digestive enzymes^92^. *PR1*, *PR2* and *PR*5 are typically involved in SA pathways, while *PR3* and *PR*4 respond to JA. The up-regulation of these *PR* genes further supports that the crosstalk between SA and JA pathways that contributes to the *Rlm1*-mediated resistance cooperatively.

### “Protein targeting to membrane”, “ribosome” and “programmed cell death”

In this study, GO enrichment analysis revealed that genes involved in “protein targeting to membrane” were significantly up-regulated in resistant samples (Fig. 4), suggesting that binding of RLKs and RLPs to plasma membrane (PM) plays a role in *Rlm1*-mediated resistance. To ensure PM functions, proteins must be properly transported from the site of synthesis, normally the endoplasmic reticulum (ER), along the secretory pathway of membrane trafficking system to PM through the Golgi apparatus and *trans-*Golgi network (TGN) by exocytosis^93,94^. As typical PM proteins, RLKs and RLPs are possibly recruited from ER upon the recognition of *Lm*.

The significant enrichment of “ribosome” among up-regulated DEGs in resistant samples (Fig. 5) may imply the contribution of ribosomal proteins to the positive regulation of *Rlm1*-mediated resistance. Ribosomes contain highly conserved proteins that function to synthesize other proteins, and their role in defense regulation has also been reported in recent years. For example, silencing the ribosomal genes *RPL12* and *RPL19* in the model plant *Nicotiana benthamiana* or Arabidopsis compromised non-host resistance to multiple bacterial pathogens^95^. Similarly, it was also found that the ribosomal protein large subunit *QM/RPL10* positively regulated defense and protein- translation mechanisms for the resistance of *N. benthamiana*^96^. The interaction of ribosomal protein *RPL12* with the receptor for activated C‐Kinase 1 (*RACK1*) also plays a key role in innate immunity in Arabidopsis^97,98^. These results support the notion that ribosomal proteins play a role in *Rlm1* resistance.

GO enrichment analysis also identified that “programmed cell death” (PCD) was suppressed in association with the resistant reaction (Fig. 4), which supports the hypothesis that host PCD contributes to host susceptibility towards fungal pathogens in necrotrophic phases. Many plants have evolved HR-associated PCD to confine the pathogen by abolishing nutrient supply, and this is particularly relevant during the process of biotrophic infection, where the cell death is generally correlated with the resistance^99,100^. In contrast, necrotrophic pathogens tend to advance infection by causing host cell death^101–104^*.*

### SAR is not induced by *Rlm1*

It was found that cotyledon inoculation, with either an avirulent or virulent *Lm* isolate, elevated SA biosynthesis and signaling in non-inoculated remote tissues (Fig. 4, Supplementary Table S4). Indeed, the *PR1* gene, which is often used as a marker for SAR, was also significantly up-regulated in these remote tissues (Supplementary Tables S2, S3). These appeared to suggest SAR, an inducible defense response that confers systemic protection against subsequent infection^46,47^. Numerous studies have reported SA as a critical component in SAR (reviewed by Conrath and Henry et al.^46,47^). Despite the elevated SA and *PR1* in remote tissues, inoculation trials (Supplementary Fig. S4) did not show substantial SAR associated with *Rlm1*; it appears that these pathways alone or the intensity to which they were activated, are insufficient to result in significant SAR. Previously, SAR was reported with the *Rlm1-*carrying canola variety ‘Columbus’ pre-inoculated with either a virulent or avirulent *Lm* isolate^84^. The discrepancy can be due to different host genetic background used; DH24288 is a DH line carries only *Rlm1* and *Rlm3* whereas it is unknown if the cultivar Columbus carries other *R* genes/QTLs which may contribute to SAR observed.

## Supplementary Information

Supplementary Information.
