# Supplementary material for: Genome-wide transcriptome reveals mechanisms underlying Rlm1-mediated blackleg resistance on canola
Source: Sci Rep. 2021 Feb 23;11:4407. doi: 10.1038/s41598-021-83267-0 (PMC7902848; doi:10.1038/s41598-021-83267-0)
Supplement: Supplementary file 1 — Supplementary Information. [file 41598_2021_83267_MOESM1_ESM.zip › Rlm1_supplementary figures_Rev1.pdf]

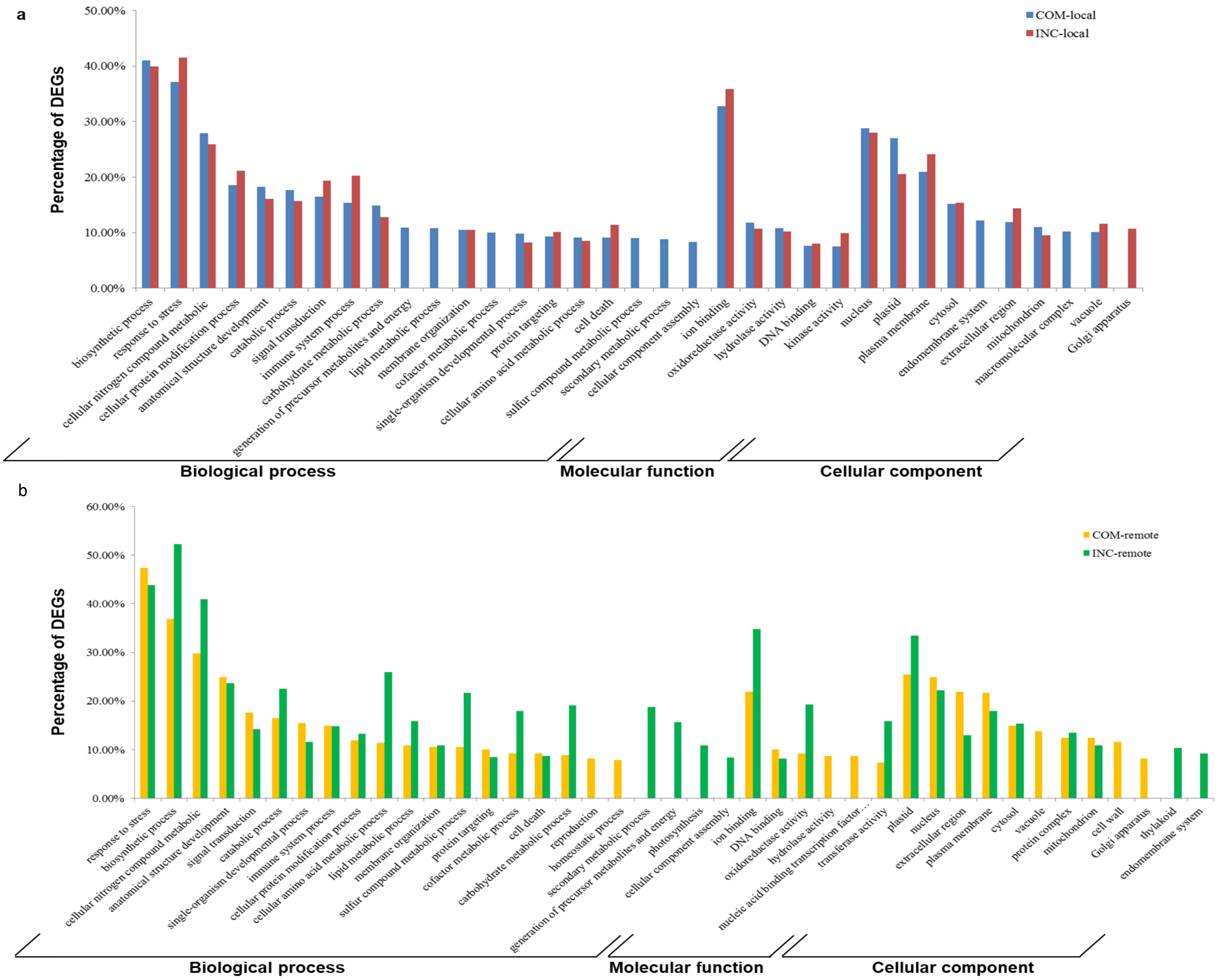

Supplementary Fig. S1. Functional classification of DEGs identified in local inoculated (a) and remote non-inoculated (b) tissues of sample plants based on GO terms**.**

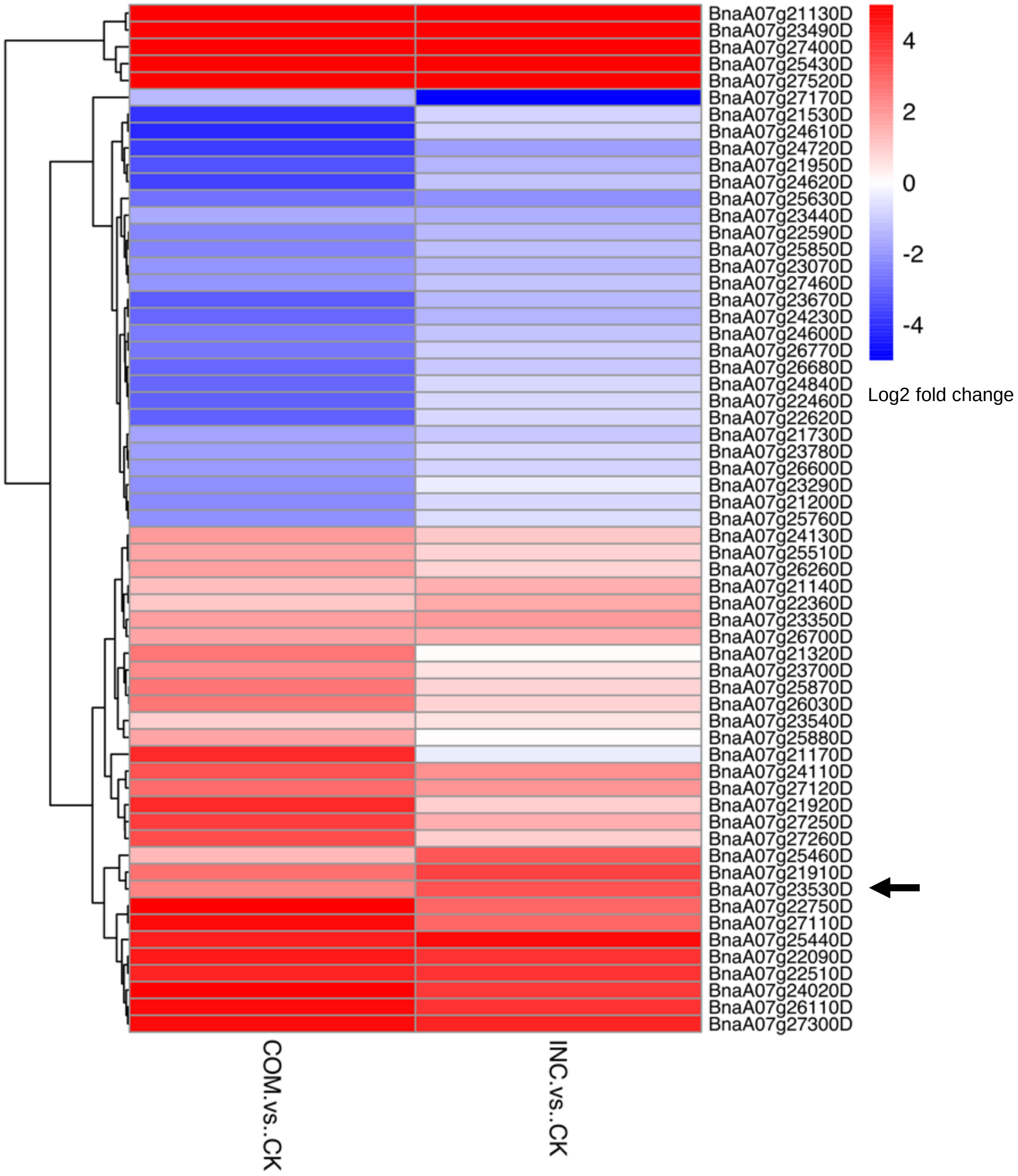

Supplementary Fig. S2. DEGs located within the *Rlm1*-mapped region.

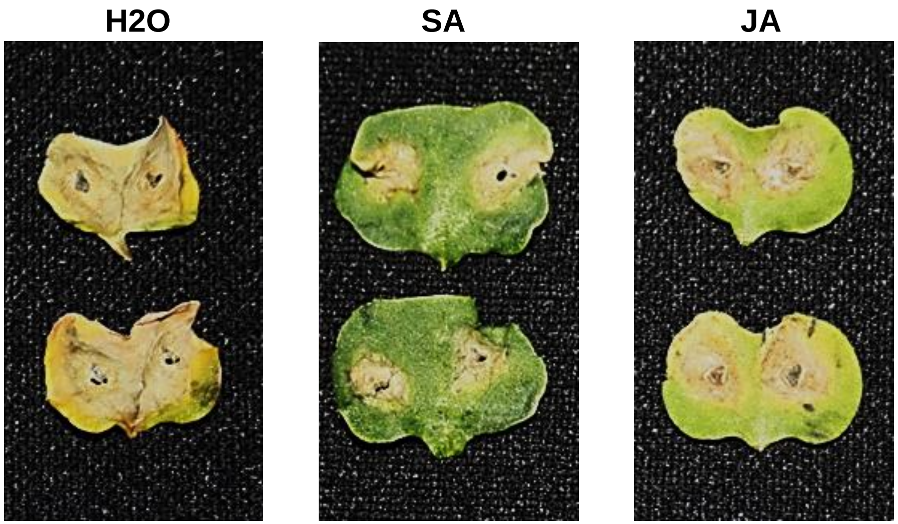

Supplementary Fig. S3. Resistance induction by exogenous phytohormone treatments of DH24288 prior to the inoculation using the virulent isolate 12CC329 (*avrLm1*).

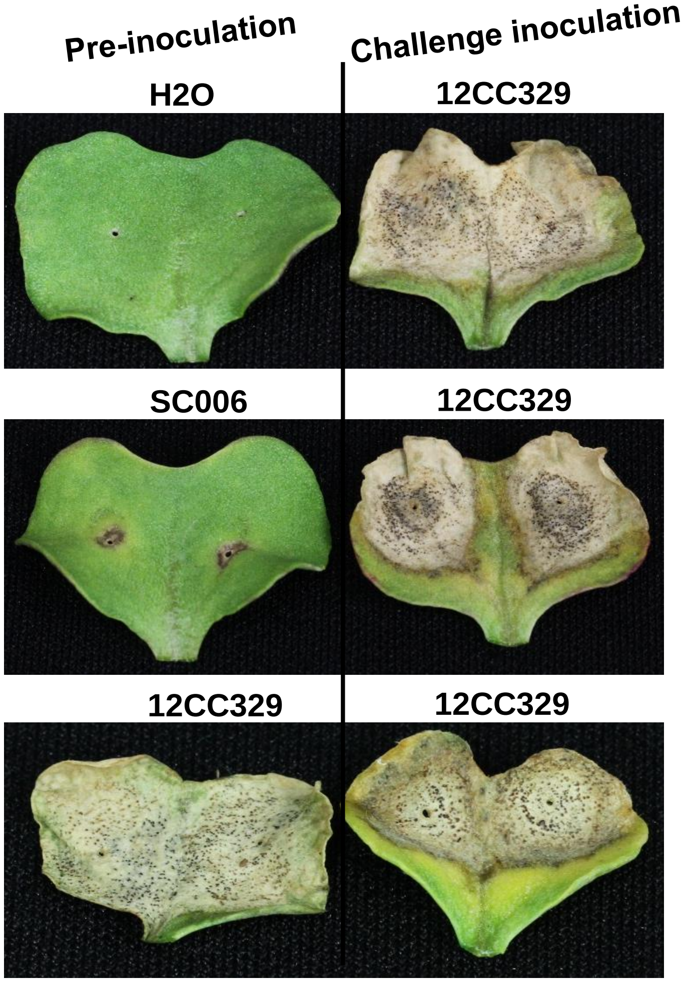

Supplementary Figure S4. Analysis of SAR on DH24288 (*Rlm1*) by pre-inoculation of one cotyledon and challenge inoculation of the other cotyledon with a virulent *L. maculans* isolates (12CC329, *avrLm*1) .
