# Supplementary material for: Genome-wide transcriptome reveals mechanisms underlying Rlm1-mediated blackleg resistance on canola
Source: Sci Rep. 2021 Feb 23;11:4407. doi: 10.1038/s41598-021-83267-0 (PMC7902848; doi:10.1038/s41598-021-83267-0)
Supplement: Supplementary file 1 — Supplementary Information. [file 41598_2021_83267_MOESM1_ESM.zip › Supplementary Table S1.docx]

Supplementary Table S1. Summary of RNA-Seq reads.

| **Statistical content** | | **Sample ID^a^** | | | | | |
| --- | --- | --- | --- | --- | --- | --- | --- |
|  |  | **COM-local** | **INC-local** | **CK-local** | **COM-remote** | **INC-remote** | **CK-remote** |
|  |  |  |  |  |  |  |  |
| Raw reads | rep 01 | 11,124,898 | 11,863,230 | 12,806,312 | 12,810,874 | 12,088,086 | 10,212,270 |
|  | rep 02 | 11,822,998 | 11,938,918 | 11,977,624 | 14,809,676 | 14,055,108 | 11,266,400 |
|  | rep 03 | 18,680,658 | 16,977,336 | 14,251,150 | 16,634,144 | 15,522,102 | 13,992,054 |
|  | total | 41,628,554 | 40,779,484 | 39,035,086 | 44,254,694 | 41,665,296 | 35,470,724 |
|  |  |  |  |  |  |  |  |
| Clean reads | rep 01 | 11,121,361 | 11,859,697 | 12,803,220 | 12,806,526 | 12,084,302 | 10,208,693 |
|  | rep 02 | 11,820,016 | 11,935,661 | 11,974,190 | 14,805,650 | 14,051,125 | 11,263,945 |
|  | rep 03 | 18,674,295 | 16,969,271 | 14,244,222 | 16,627,303 | 15,514,709 | 13,984,477 |
|  | total | 41,615,672 | 40,764,629 | 39,021,632 | 44,239,479 | 41,650,136 | 35,457,115 |
|  |  |  |  |  |  |  |  |
| Mapped reads | rep 01 | 6,339,746 | 8,875,902 | 9,657,698 | 9,781,170 | 9,261,367 | 7,786,060 |
|  | rep 02 | 6,463,974 | 9,021,700 | 9,102,347 | 11,404,128 | 10,695,572 | 8,395,3393 |
|  | rep 03 | 10,670,927 | 12,737,405 | 11,019,567 | 12,656,200 | 11,813,561 | 10,402,725 |
|  | total (%) | 23,474,647 (56.4) | 30,635,007 (75.1) | 29,679,612 (76.0) | 33,841,498 (76.5) | 31,770,500 (76.3) | 26,584,178 (75.0) |
|  |  |  |  |  |  |  |  |
| Uniquely mapped reads | rep 01 | 5,882,664 | 8,215,910 | 8,880,587 | 9,065,325 | 8,561,163 | 7,219,865 |
|  | rep 02 | 5,993,050 | 8,341,594 | 8,314,403 | 10,567,088 | 9,889,235 | 7,794,246 |
|  | rep 03 | 9,882,970 | 11,790,250 | 10,177,654 | 11,735,936 | 10,941,339 | 9,655,490 |
|  | total (%) | 21,758,684 (52.3) | 28,347,754 (69.5) | 27,372,644 (70.1) | 31,368,349 (70.9) | 29,391,737 (70.6) | 24,669,701 (69.6) |
|  |  |  |  |  |  |  |  |
| Multiple mapped reads | rep 01 | 457,082 | 659,992 | 677,111 | 715,845 | 806,337 | 566,195 |
|  | rep 02 | 470,924 | 680,106 | 787,944 | 837,040 | 700,204 | 601,047 |
|  | rep 03 | 787,957 | 947,155 | 841,913 | 920,264 | 872,222 | 747,235 |
|  | total (%) | 1,715,963 (4.1) | 2,287,253 (5.6) | 2,306,968 (5.9) | 2,473,149 (5.6) | 2,378,763 (5.7) | 1,914,477 (5.4) |
|  |  |  |  |  |  |  |  |
| Unmapped reads | rep 01 | 4,781,615 | 2,983,795 | 3,245,522 | 3,025,356 | 2,822,935 | 2,422,633 |
|  | rep 02 | 5,356,042 | 2,913,961 | 2,871,843 | 3,401,522 | 3,355,553 | 2,868,552 |
|  | rep 03 | 8,003,368 | 4,231,866 | 3,224,655 | 3,971,103 | 3,701,148 | 3,581,752 |
|  | total (%) | 18,141,025 (43.6) | 10,129,622 (24.9) | 9,342,020 (24.0) | 10,397,981 (23.5) | 9,879,636 (23.7) | 8,872,937 (25.0) |
|  |  |  |  |  |  |  |  |
| Unique genes |  | 58,700 | 60,563 | 61,417 | 60,754 | 60,179 | 60,485 |

^a^COM-local, INC-local, and CK-local: samples from the inoculated cotyledons of the plants treated with 12CC329, SC006 or water, respectively; COM-remote, INC- remote, and CK- remote: samples from the corresponding non-inoculated cotyledons of the same plants.
