# Supplementary material for: Genome-wide transcriptome reveals mechanisms underlying Rlm1-mediated blackleg resistance on canola
Source: Sci Rep. 2021 Feb 23;11:4407. doi: 10.1038/s41598-021-83267-0 (PMC7902848; doi:10.1038/s41598-021-83267-0)
Supplement: Supplementary file 1 — Supplementary Information. [file 41598_2021_83267_MOESM1_ESM.zip › Supplementary Table S7.docx]

Supplementary Table S6. Gene information and primer sequence for qRT-PCR validation.

| **Gene ID** | **Annotation** | **Primers (5’ to 3’)** |
| --- | --- | --- |
| BnaA01g00580D | GH3 | F: ACAAGCTCTGGGACATCTGC  R: AAAAGCTGACGCCGGTCTAA |
| BnaA01g20340D | EDS1 | F: TGAAGAACGAAGACACGGGG  R: GCGTCTGTGAACCTCAGGAA |
| BnaA03g20310D | CHI | F: TGCTACATAGAAGAAATAAACGG  R: TTCCATGATAGTTGAATCGG |
| BnaA03g28770D | PR4 | F: ACTGGGATTTGAGAGCCGTG  R: CTCCGCCACGAGTATGGTTT |
| BnaA03g38630D | PR1 | F: CATCCCTCGAAAGCTCAAGAC  R: CCACTGCACGGGACCTAC |
| BnaA07g32130D | PDF2.1 | F: ATGGCTAAGTTTGTTTCCATCA  R: ACTCCTGACCATGTCCCACT |
| BnaA09g35840D | WRKY70 | F: ACATACATAGGAAACCACACG  R: ACTTGGACTATCTTCAGAATGC |
| BnaA09g45360D | PCR2 | F: GCATTACCGTTGGCCGAATC  R: ATAGCATGTACAGCGCACCA |
| BnaA09g50590D | AXR3 | F: ACCTGTGAGGTCATACCGGA  R: CGTCCATCGACACCTTCACA |
| BnaC02g08080D | PK | F: GTCGGTTTCCGCCTTTTCAC  R: GAGTGAAGACACCACCCGAG |
| BnaC06g33300D | Protease inhibitor | F: CCACGGCACTACAGAATCCA  R: CTCTGTCTTAACCGGCAGCA |
| BnaC07g29940D | ACO | F: TTTACCTGCTCACACCGACC  R: TGTGGATTAACGGCGAACCA |
| BnaC08g49310D | ADH | F: CATGGAGCTCTTCCAAGGCA  R: CCTTGGAGGCACTGTGTGAA |
| BnaCnng07500D | PR2 | F: TGGGTCAAACGCATACCGAA  R: GCCAACCACTCTCTGAGACC |
| BnaUnng02510D | AAO | F: TGCCTCTGGCAAGGATCAAG  R: TTTTGCCCACCTTTGGGGAT |
| BnaCnng32080D | NCED3 | F: CGATTTGCCTTACCAAGTCAG  R: TTTATCCCTTCCGGTGAGAA |
