# Supplementary material for: Genome-wide transcriptome reveals mechanisms underlying Rlm1-mediated blackleg resistance on canola
Source: Sci Rep. 2021 Feb 23;11:4407. doi: 10.1038/s41598-021-83267-0 (PMC7902848; doi:10.1038/s41598-021-83267-0)
Supplement: Supplementary file 1 — Supplementary Information. [file 41598_2021_83267_MOESM1_ESM.zip › Title page.docx]

Genome-wide transcriptome reveals mechanisms underlying *Rlm1-*mediated blackleg resistance on canola

Chun Zhai, Xiujia Liu, Tao Song^1^, Fengqun Yu, Gary Peng *

Agriculture and Agri-Food Canada (AAFC), Saskatoon Research and Development Centre, Saskatoon, Saskatchewan, Canada

^1^ Current address: Syngenta Biotechnology China Co., Ltd, Beijing, China

* Correspondence to: [gary.peng@canada.ca](mailto:gary.peng@canada.ca)
